# Functional Characterization of Newly-Discovered Mutations in Human SR-BI

**DOI:** 10.1371/journal.pone.0045660

**Published:** 2012-09-21

**Authors:** Alexandra C. Chadwick, Daisy Sahoo

**Affiliations:** 1 Department of Pharmacology and Toxicology, Medical College of Wisconsin, Milwaukee, Wisconsin, United States of America; 2 Department of Medicine, Division of Endocrinology, Metabolism and Clinical Nutrition, Medical College of Wisconsin, Milwaukee, Wisconsin, United States of America; Harvard Medical School, United States of America

## Abstract

In rodents, SR-BI has been firmly established as a physiologically relevant HDL receptor that mediates removal of HDL-cholesteryl esters (CE). However, its role in human lipoprotein metabolism is less defined. Recently, two unique point mutations in human SR-BI — S112F or T175A — were identified in subjects with high HDL-cholesterol (HDL-C) levels. We hypothesized that mutation of these conserved residues would compromise the cholesterol-transport functions of SR-BI. To test this hypothesis, S112F- and T175A-SR-BI were generated by site-directed mutagenesis. Cell surface expression was confirmed for both mutant receptors in COS-7 cells upon transient transfection, albeit at lower levels for T175A-SR-BI. Both mutant receptors displayed defective HDL binding, selective uptake of HDL-CE and release of free cholesterol (FC) from cells to HDL. Mutant receptors were also unable to re-organize plasma membrane pools of FC. While these impaired functions were independent of receptor oligomerization, inability of T175A-SR-BI to mediate cholesterol-transport functions could be related to altered N-linked glycosylation status. In conclusion, high HDL-C levels observed in carriers of S112F- or T175A-SR-BI mutant receptors are consistent with the inability of these SR-BI receptors to mediate efficient selective uptake of HDL-CE, and suggest that increased plasma HDL concentrations in these settings may not be associated with lower risk of cardiovascular disease.

## Introduction

The reverse cholesterol transport (RCT) pathway, whereby high density lipoproteins (HDL) transport cholesterol from peripheral tissues to the liver for catabolism [1], is critical for combating coronary artery disease since it prevents the accumulation of excess cholesterol, lipids, and cellular debris, and thus the formation of atherosclerotic plaques on arterial walls [2]. In the last step of RCT, HDL binds to scavenger receptor class B type I (SR-BI), an 82 kDa glycosylated cell-surface receptor that mediates selective uptake of HDL-cholesteryl esters (CE) into the liver [3], without holo-particle uptake [4]–[6]. SR-BI also stimulates free cholesterol (FC) efflux from peripheral tissues to HDL [7], although its role in stimulating this early step of RCT to decrease macrophage foam cell formation remains controversial [8], [9].

The selective uptake of HDL-CE is only achieved if HDL and SR-BI form a “productive complex”, where the receptor and ligand precisely align to allow CE transfer to occur [10]. Previous studies have validated the importance of SR-BI's extracellular domain in the selective uptake process through use of chimeric receptors [11], [12], epitope tag insertion [13], mutagenesis [14], [15], and blocking antibody directed against the extracellular domain [16]. Further, our recent work demonstrates that hydrophobicity of the N-terminal half of the extracellular domain of SR-BI [15], as well as a specific conformation of the C-terminal half of the extracellular domain held together by disulfide bonds [17], are critical for receptor function.

Transgenic overexpression [18]–[20] or hepatic adenoviral-mediated [21], [22] SR-BI cDNA transfer in mice decreased plasma HDL-cholesterol (HDL-C) levels and increased cholesterol catabolism and excretion. On the other hand, reduced SR-BI expression or whole-body knock-out of the SR-BI gene resulted in increased plasma HDL-C levels [23]. Although these studies have firmly established SR-BI as a physiologically relevant HDL receptor in rodents, its role in human lipoprotein metabolism is less defined. The human homologue of SR-BI, also known as CLA-1 (CD36 and LIMPII analogous-1), serves as a receptor for HDL and mediates HDL-CE selective uptake [24]. Similar to rodents, it is also most abundantly expressed in the liver and steroidogenic tissues [24]. Human SR-BI variants are associated with changes in HDL-cholesterol [25], [26] and protein levels in peripheral tissues [27]. Most recently, two point mutations in human SR-BI — at Ser112 (to Phe) or at Thr175 (to Ala) — were identified in two subjects with high HDL-C levels [28]. As these are highly conserved residues across species, we predicted that SR-BI's function would be compromised.

In this study, we characterized the functionality of these SR-BI variants in COS-7 cells to test our hypothesis that high HDL-cholesterol levels in subjects harboring mutations at Ser112 or Thr175 result from the inability of SR-BI to mediate efficient HDL-CE selective uptake.

## Methods

### Materials

The following antibodies were used: anti-SR-BI specific for the extracellular domain or the C-terminal cytoplasmic domain (Novus Biologicals, Inc., Littleton, CO), anti-glyceraldehyde-3-phosphate dehydrogenase (GAPDH) (Millipore, Billerica, MA), peroxidase-conjugated goat anti-rabbit IgG (Jackson ImmunoResearch Laboratories, West Grove, PA). Human HDL was purchased from Biomedical Technologies, Inc. [^3^H]Cholesteryl oleoyl ether (COE) was purchased from American Radiolabeled Chemicals, Inc (St. Louis, MO). [^125^I]Sodium iodide and [^3^H]cholesterol were purchased from Perkin-Elmer. Cholesterol oxidase (*Streptomyces*), cholesterol, 4-cholesten-3-one, and cholesteryl oleate standards were purchased from Sigma. EZ-Link Sulfo-NHS-LC-biotin was purchased from Thermo Fisher Scientific (Rockford, IL). All other reagents were of analytical grade.

### Plasmids

The human SR-BI coding region was cloned into the pcDNA3.1 vector (Invitrogen) to produce pcDNA3.1[hSR-BI] (herein referred to as SR-BI). Site-directed mutations at Ser112 (to Phe) or Thr175 (to Ala) were then introduced into pcDNA3.1[hSR-BI]. Cloning, mutagenesis and sequencing were performed by Top Gene Technologies (Pointe-Claire, Quebec, Canada).

### Cell Culture and Transfection

COS-7 cells, maintained in DMEM (Invitrogen) containing 10% calf serum (Invitrogen), 2 mM _L_-glutamine, 50 units/mL penicillin, 50 µg/mL streptomycin, and 1 mM sodium pyruvate, were transiently transfected with Fugene 6 as previously described [29]. Unless otherwise noted, cellular assays were performed 48 hours post-transfection.

### Cell lysis

Transiently-transfected COS-7 cells were washed twice with cold PBS (pH 7.4) and lysed with 1% NP-40 cell lysis buffer containing protease inhibitors. Protein concentrations were determined by the Lowry method as previously described [30].

### Cell surface receptor expression

Cell surface biotinylation of SR-BI in transiently-transfected COS-7 cells was performed as previously described [15]. In separate experiments, cell surface expression of SR-BI was verified by flow cytometry as described using FACS Calibur (Flow Cytometry Core, Medical College of Wisconsin) [17].

### HDL labeling, cell association of [^125^I]HDL and uptake of [^3^H]HDL-COE

HDL was double-labeled with non-hydrolyzable [^3^H]COE and [^125^I]dilactitol tyramine as described [29]. Preparations of radiolabeled HDL had an average [^3^H] specific activity of 220.83 dpm/ng of protein and an average [^125^I] specific activity of 280.89 dpm/ng of protein. COS-7 cells transiently transfected with empty pcDNA3.1 vector, wild-type or mutant human SR-BI were assayed for cell association of [^125^I]-HDL and selective uptake of non-hydrolyzable [^3^H]-COE as previously described [29]. Statistical analysis was determined by one-way ANOVA.

### Free cholesterol efflux and cholesterol oxidase sensitivity

Cells transiently transfected with cDNA encoding empty vector, wild-type or mutant human SR-BI were pre-labeled with [^3^H]cholesterol and assayed for FC release from cells to HDL, or for sensitivity to exogenous cholesterol oxidase at 72-hours or 48- hours post-transfection, respectively, as previously described [13]. Statistical analysis was determined by one-way ANOVA.

### SR-BI oligomerization

Cells transiently expressing wild-type and mutant SR-BI were lysed in PBS containing protease inhibitors. SR-BI oligomerization was assessed by 6% PFO-PAGE as previously described [15].

## Results

### Variable cell surface expression of mutant SR-BI receptors

**Figure 1:**
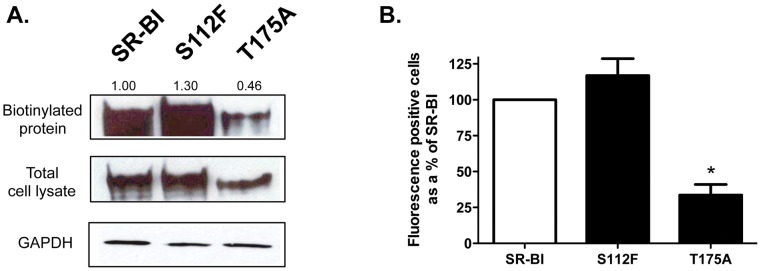
Cell surface expression of wild-type and mutant SR-BI receptors. (A) COS-7 cells expressing wild-type or mutant SR-BI receptors were assessed for cell surface expression following incubation with NHS-LC-Biotin as described in Materials and Methods. Immunoblot analyses of biotinylated SR-BI at the cell surface (from ∼150 µg of total lysate) (top panel) and in 20 µg of total cell lysate (middle panel) are shown using an antibody directed against the C-terminal cytoplasmic domain of SR-BI. GAPDH was detected as a loading control (bottom panel). The numbers above the top panel represent cell surface receptor expression by densitometry analysis (where SR-BI = 100%). Data are representative of 3 independent experiments. (B) Surface expression of wild-type or mutant SR-BI receptors in COS-7 cells was assessed by flow cytometry using an antibody directed against the extracellular domain of SR-BI. Data are expressed as a % of SR-BI expression following subtraction of empty vector values. Data are the average of 12 independent transfections.

Ser112 and Thr175 are evolutionarily conserved residues. Using site-directed mutagenesis, we created S112F and T175A point mutations in human SR-BI to test their ability to mediate cholesterol-transport functions. We chose to study SR-BI function in transfected COS-7 cells since they do not have endogenous SR-BI expression (as confirmed by immunoblot and flow cytometry analyses; [31] and data not shown). We used two different methods to assess cell surface expression of both SR-BI mutants. In the first method, biotinylation and immunoblot analysis of COS-7 cell lysates revealed that wild-type and S112F-SR-BI expressed at approximately similar levels at the cell surface. However, T175A-SR-BI expressed more than 50% less than wild-type SR-BI based on band densitometry (**Figure 1A** **, top panel**). Total cell expression of all SR-BI constructs are shown in **Figure 1A** **(middle panel)**. These observations were mirrored in parallel experiments where flow cytometry analyses also revealed that T175A-SR-BI expressed at much lower levels at the cell surface than wild-type or S112F-SR-BI (**Figure 1B**).

### Mutant receptors displayed an impaired ability to bind HDL and mediate selective uptake of HDL-COE

**Figure 2:**
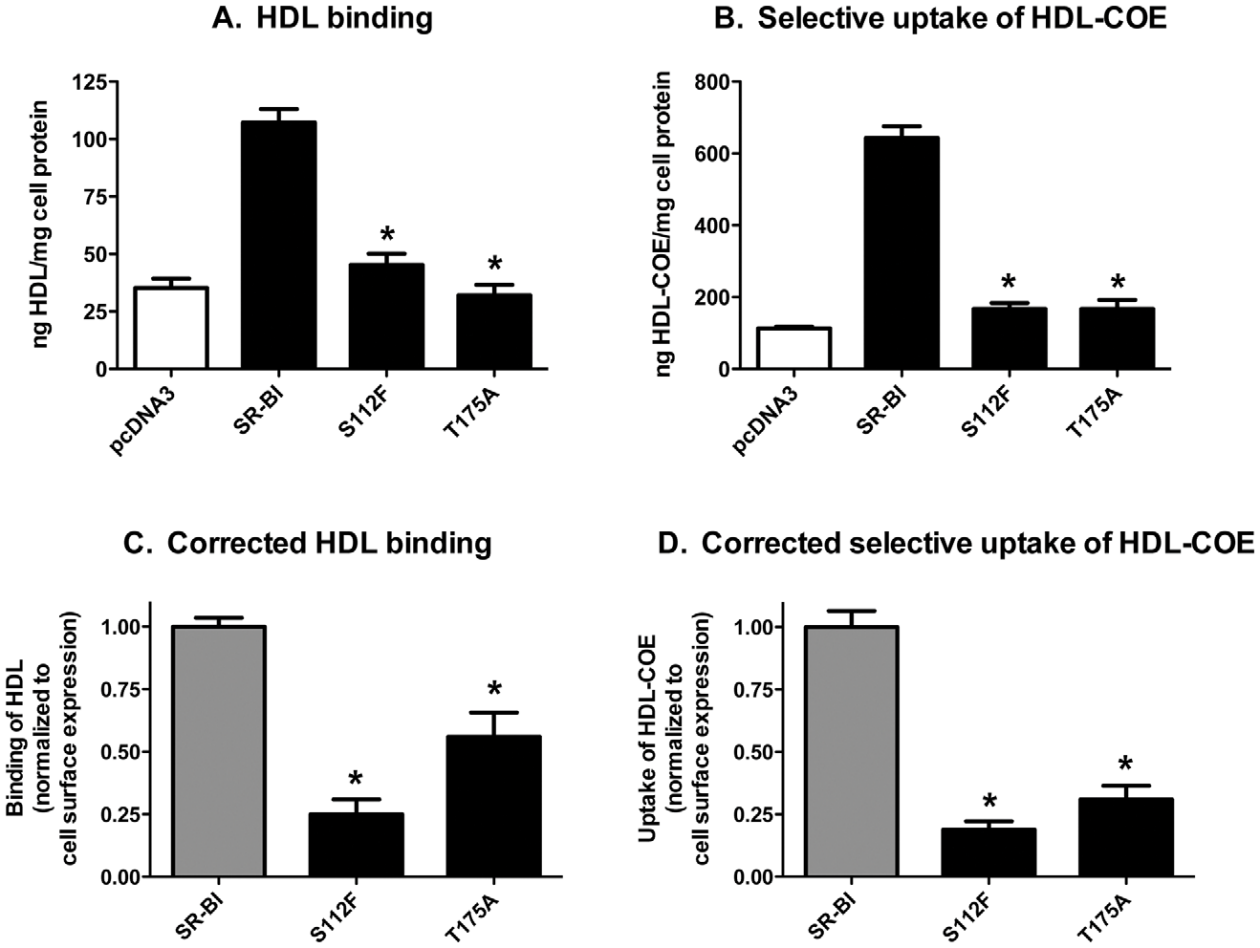
Mutant SR-BI receptors display decreased HDL binding and selective uptake of HDL-COE. COS-7 cells transiently expressing wild-type or mutant SR-BI were incubated with [^125^I]/[^3^H]-COE-labeled HDL (10 µg HDL protein/mL). (A) Binding of [^125^I]HDL and (B) selective uptake of [^3^H]COE are shown. (C, D) Individual data sets from panels A and B were first normalized to wild-type SR-BI (normalized value = 100%) following subtraction of empty vector values. Next, the corrected HDL binding and HDL-COE values (panels C and D, respectively) were calculated by dividing the normalized values for each receptor by the corresponding value for surface expression (by flow cytometry) from parallel wells within the same experiment. Combined data from six independent experiments, each performed in triplicate, are shown. *p<0.001, as determined by one-way ANOVA.

To determine if mutant SR-BI receptors shared similar functional properties as wild-type SR-BI, we assessed the ability of all SR-BI receptors to bind HDL and mediate the selective uptake of HDL-COE by transiently transfecting COS-7 cells with vectors encoding mutant SR-BI proteins. As shown in **Figure 2A**, S112F- and T175A-SR-BI displayed significantly lower levels of HDL binding as compared to wild-type SR-BI, even after normalization to cell surface expression based on flow cytometry analyses (**Figure 2C**) from parallel wells from the same experiments. As expected, the inability of both mutants to properly bind HDL translated to an impaired ability to mediate efficient selective uptake of HDL-COE (**Figures 2B, D**).

### Mutant receptors display decreased efflux of free cholesterol to HDL

**Figure 3:**
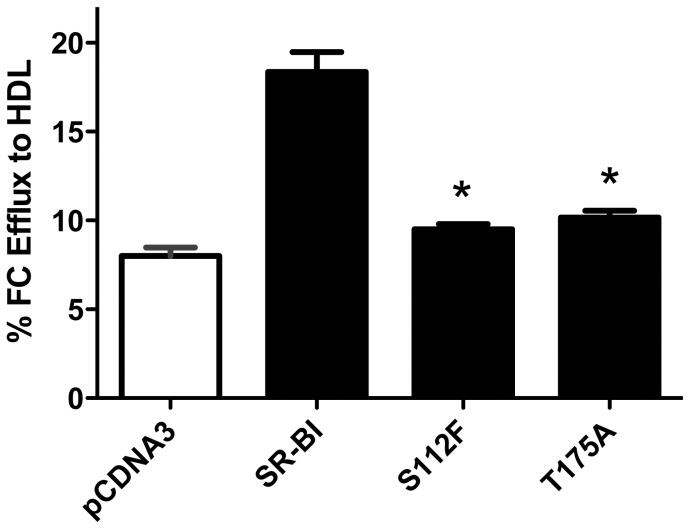
Mutant SR-BI receptors are unable to mediate efflux of free cholesterol from cells to HDL. COS-7 cells transiently expressing wild-type or mutant SR-BI and pre-labeled with [^3^H]-cholesterol were incubated with HDL (50 µg/mL) for four hours. Cell and media radioactivity were assessed. Combined data from four independent experiments, each performed in quadruplicate, are shown. *p<0.001, as determined by one-way ANOVA.

In addition to its role in selective uptake of HDL-CE, SR-BI also plays a role in stimulating the transfer of FC from peripheral cells to acceptor particles such as HDL [3]. We measured the ability of wild-type, S112F- and T175A-SR-BI receptors to efflux FC from pre-labeled COS-7 cells to HDL acceptors. As shown in **Figure 3**, both the S112F- and T175A-SR-BI mutant receptors displayed significant decreases in FC efflux as compared to wild-type SR-BI. As expected [32]–[35], little to no wild-type- or mutant SR-BI-mediated FC efflux was observed when lipid-free apoA-I was used as an acceptor (data not shown), thus suggesting that mutant SR-BI receptors affect intracellular and/or membrane pools of FC only in the presence of HDL.

### Mutant SR-BI receptors are unable to re-organize plasma membrane free cholesterol

**Figure 4:**
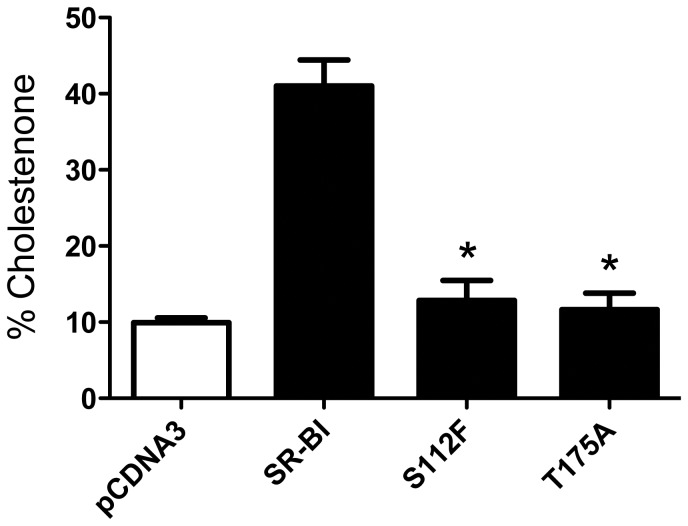
Mutant receptors are unable to re-organize plasma membrane pools of FC. COS-7 cells transiently expressing wild-type or mutant SR-BI and pre-labeled with [^3^H]-cholesterol were incubated with cholesterol oxidase (0.5 units/mL) for four hours. Lipids were extracted and radioactivity associated with cholestenone was assessed by thin layer chromatography. Combined data from four independent experiments, each performed in quadruplicate, are shown. *p<0.001, as determined by one-way ANOVA.

Native SR-BI plays a unique role in re-organizing the pools of plasma membrane FC as judged by the enhanced conversion of cholesterol to cholestenone in the presence of exogenous cholesterol oxidase [36]. Quantification of membrane-associated cholestenone following incubation with exogenous cholesterol oxidase revealed that both the S112F- and T175A-SR-BI mutant receptors displayed significantly decreased abilities to re-distribute plasma membrane content of FC as compared to wild-type SR-BI (**Figure 4**).

### Defective cholesterol transport functions of SR-BI mutants are not due to changes in their oligomeric status

**Figure 5:**
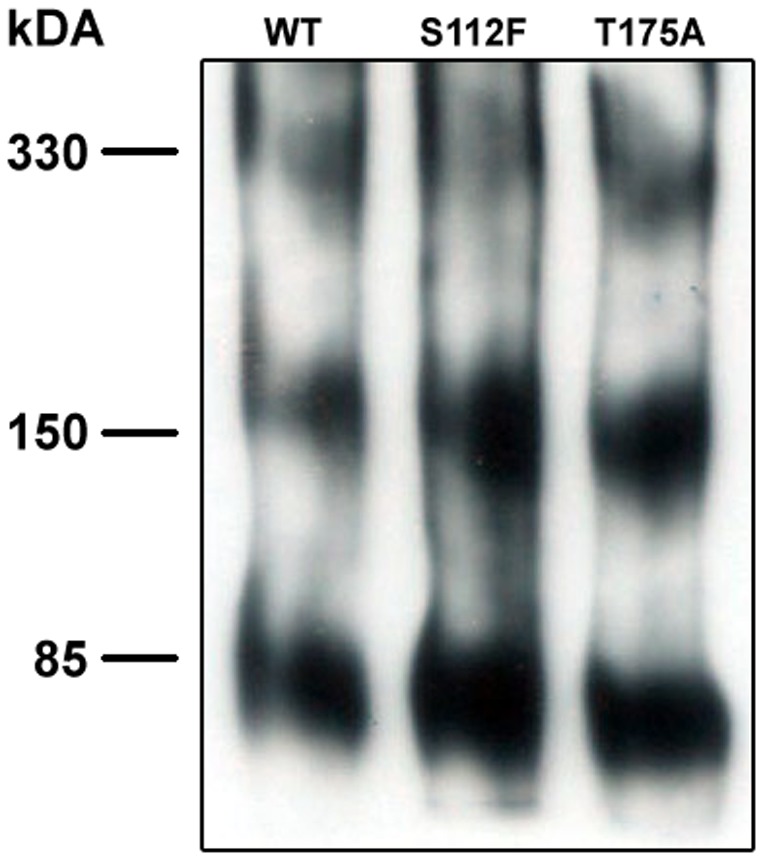
Mutant receptors maintain their ability to form homo-oligomers. COS-7 cell lysates transiently expressing wild-type or mutant SR-BI were separated by 6% PFO-PAGE. SR-BI was detected by immunoblot analysis using an antibody directed against the C-terminal domain of the receptor. Data is representative of 4 independent experiments.

SR-BI exists as dimers and higher-order oligomers at the plasma membrane [37]–[39]. We and others suggest that SR-BI oligomerization is required for efficient selective uptake of HDL-CE [17], [39], [40], lending support to the notion that SR-BI oligomers form a hydrophobic channel to allow transfer of CE from HDL into the plasma membrane [41]. To determine whether the inability of the S112F- and T175A-SR-BI mutant receptors to mediate proper cholesterol-transport functions was due to their inability to form higher-order oligomers, we assessed their oligomeric status by performing native gel electrophoresis in the presence of perfluorooctanoic acid (PFO), a detergent known to stabilize interactions between oligomers [15], [17], [42]. Based on our analyses, both the S112F- and T175A- mutant receptors were able to form higher order oligomers similar to wild-type SR-BI (**Figure 5**). These data suggest that the impaired function exhibited by the human SR-BI mutations is independent of the oligomeric status of the receptors.

### T175A-SR-BI may be impaired due to altered glycosylation patterns

**Figure 6:**
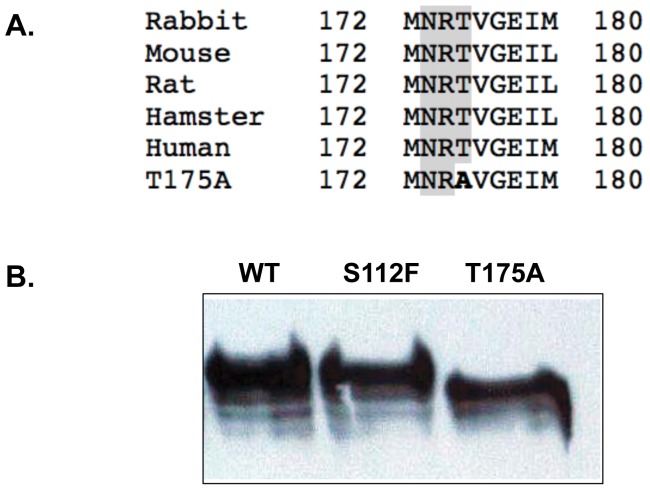
T175A-SR-BI exhibits an altered glycosylation pattern. (A) The evolutionarily-conserved consensus sequence for N-linked glycosylation is shaded in gray for rabbit (AY_283277), mouse (NM_016741), rat (NP_113729.1), hamster (A53920) and human (NM_005505) SR-BI. The Thr-to-Ala mutation at residue 175 (in bold) disrupts this consensus sequence. (B) COS-7 cell lysates transiently expressing wild-type or mutant SR-BI were separated by 12% SDS-PAGE. SR-BI was detected by immunoblot analysis using an antibody directed against the C-terminal cytoplasmic domain of the receptor. Data is representative of 7 independent experiments.

Residue 175 of human SR-BI is part of an N-linked glycosylation sequence (NXS/T) [43], [44] that is highly conserved among species (**Figure 6A**). Mutation of Thr175 to Ala disrupts proper glycosylation of Asn173 as evidenced by a faster mobility on SDS-PAGE as compared to wild-type SR-BI or the S112F-SR-BI mutant receptor (**Figure 6B**). This data suggests that the lower cell surface expression and impaired function of T175A-SR-BI may be the result of an altered glycosylation status of the receptor.

## Discussion

The recent identification of two novel missense mutations, S112F and T175A in human SR-BI, prompted us to characterize the functionality of these two variants of SR-BI that underlie elevated HDL-cholesterol levels in humans. Our data revealed that both mutant SR-BI receptors had significantly impaired abilities to bind HDL and mediate the selective uptake of HDL-COE. In addition to displaying defective efflux of FC to HDL, both mutants were also unable to redistribute the plasma membrane pools of FC. The impaired functions of these mutant receptors were not due to changes in oligomeric status. However, decreased cell surface expression and defective functions of T175A-SR-BI could be explained, in part, by an altered glycosylation status as compared to wild-type SR-BI.

The accumulation of HDL-cholesterol levels upon deletion of the SR-BI gene in mice [23] strongly suggests that the athero-protective role of SR-BI [45]–[47] is primarily due to its ability to mediate the selective uptake of CE from circulating HDL particles [23], [48], [49]. Based on our in vitro studies, we suggest that inefficient clearance of HDL-C from circulation may explain the 37% increases in HDL-C (with no significant differences in triglycerides, LDL-cholesterol or body mass index) in the two individuals heterozygous for mutations at Ser112 or Thr175 compared to family member controls [28]. As carriers of P297S-SR-BI, the first human SR-BI mutation, displayed similar increases in HDL-C levels [50], our data lend support to the notion that human SR-BI does indeed play a role in hepatic HDL-CE uptake.

The athero-protective effects of SR-BI can also be explained, in part, by its role in mediating FC efflux from lipid-laden macrophages to HDL, although this remains controversial [8], [51]–[54]. The reduced ability of S112F- and T175A-SR-BI mutant receptors to efflux FC to HDL mirrors similar trends observed with the P297S-SR-BI mutant receptor in mice [50]. At least in the case of T175A-SR-BI, lower levels of FC efflux may correlate with its lower levels of expression at the cell surface [7], [55]. The inability of both mutant receptors to efficiently mediate cholesterol-transport functions may be associated, in part, with their inability to mediate plasma membrane reorganization of cholesterol, as we have previously observed with other SR-BI receptors harboring mutations in this region of the extracellular domain [15]. More in-depth cholesterol-transport and membrane re-organization studies in macrophage or hepatocyte/hepatoma cell lines warrant further investigation and can provide sharper insight as to how these mutant SR-BI receptors behave when all efflux machinery (i.e. ABCA1 and ABCG1) is present.

Mutation of Thr175 to alanine appears to disrupt N-linked glycosylation at the evolutionarily-conserved Asn173 residue as judged by its faster migration as compared to the wild-type or S112F-SR-BI receptors following SDS-PAGE. It was previously demonstrated that N-linked oligosaccharide chains at Asn173 are a critical determinant of cell surface expression and efficient lipid uptake [43]. In support of these observations, reduced cell surface expression of T175-SR-BI similarly correlates with reduced selective HDL-CE uptake efficiency. Although oligosaccharides on glycoproteins have been shown to participate in protein-protein interactions in other systems [56], [57], our analyses indicated that SR-BI oligomerization is not affected by altered glycosylation status. Rather, it is likely that altered glycosylation in T175A-SR-BI and subsequent changes in cell surface expression result from either improper folding in the ER and consequent transport through the Golgi, or decreased receptor stability [43].

Mutation of Ser112 to Phe did not affect cell surface receptor expression, although it did significantly affect HDL-CE selective uptake efficiency. Interestingly, Ser112 lies adjacent to another N-linked glycosylation site (Asn108) that is critical for cell surface expression and function [43]. It is certainly possible that introduction of a large amino acid like phenylalanine could impede the addition of oligosaccharides at Asn108, however an altered glycosylation status S112F-SR-BI was not evident from our immunoblot analyses. Alternatively, proper receptor-ligand interactions may be compromised by (i) altered secondary or tertiary structure of the area surrounding Ser112, (ii) the large nature of the phenyl side chain [58], or (iii) the inability of phenylalanine to participate in potential hydrogen bonding with residues on HDL proteins due to the lack of a hydroxyl group [59]–[61]. Remarkably, both the S112F and T175A point mutations occur in conserved regions of hydrophobicity in the N-terminal half of the extracellular domain of SR-BI. We have previously shown that the hydrophobicity of this region is critical for SR-BI-mediated cholesterol transport function [15] and hypothesized that this region may interact with the plasma membrane as has been reported for other integral membrane proteins [62], [63]. While mutation of Thr to Ala does not impose major effects on hydrophobicity, mutation of Ser to Phe (hydrophobic index of −0.8 and 2.8, respectively [64]) significantly increases the hydrophobicity of the region surrounding residue 112. This leads us to speculate that the region surrounding the Phe residue becomes less solvent-exposed, and perhaps even more ‘buried’ in the membrane, thus preventing key conformational changes that may be required to support efficient selective uptake of HDL-CE. These ideas are currently being investigated.

Although human and rodent SR-BI share similar expression patterns, tissue abundance and in vitro receptor activities [3], [24], [38], [65], the role of human SR-BI in hepatic clearance of HDL-CE and its relevance to human physiology has remained a mystery until recently. Not surprisingly, our functional characterization of these two newest human SR-BI mutations raises new questions. Due to inefficient clearance of plasma HDL particles, do carriers of the S112F or T175A mutations possess larger-sized HDL particles, similar to carriers of the P297S mutation [50], and SR-BI knock-out mice [23], [50]? Has the oxidation status and efflux capacity of circulating HDL (i.e. HDL ‘functionality’) been altered? Importantly, carriers of SR-BI mutations at Ser112, Thr175 or Pro297 do not currently show clinical signs of atherosclerosis. Therefore, identification and investigation of additional subjects with the same or new SR-BI mutations will be invaluable as we address the on-going debate as to whether higher HDL-C levels are truly athero-protective [66]–[69]. Answers to these lingering questions are particularly relevant in light of current efforts directed at developing HDL-raising therapeutics to lower cardiovascular risk [70], [71].

## References

[1] Glomset JA (1968) The plasma lecithins:cholesterol acyltransferase reaction. J Lipid Res 9: 155–167. 4868699

[2] Glass CK, Witztum JL (2001) Atherosclerosis. the road ahead. Cell 104: 503–516. 11239408 10.1016/s0092-8674(01)00238-0

[3] Acton S, Rigotti A, Landschulz KT, Xu S, Hobbs HH, et al (1996) Identification of scavenger receptor SR-BI as a high density lipoprotein receptor. Science 271: 518–520. 8560269 10.1126/science.271.5248.518

[4] Glass C, Pittman RC, Civen M, Steinberg D (1985) Uptake of high-density lipoprotein-associated apoprotein A-I and cholesterol esters by 16 tissues of the rat in vivo and by adrenal cells and hepatocytes in vitro. J Biol Chem 260: 744–750. 3918032

[5] Pittman RC, Glass CK, Atkinson D, Small DM (1987) Synthetic high density lipoprotein particles. Application to studies of the apoprotein specificity for selective uptake of cholesterol esters. J Biol Chem 262: 2435–2442. 3029080

[6] Pittman RC, Knecht TP, Rosenbaum MS, Taylor CA Jr (1987) A nonendocytotic mechanism for the selective uptake of high density lipoprotein-associated cholesterol esters. J Biol Chem 262: 2443–2450. 2434485

[7] Ji Y, Jian B, Wang N, Sun Y, Moya ML, et al (1997) Scavenger receptor BI promotes high density lipoprotein-mediated cellular cholesterol efflux. J Biol Chem 272: 20982–20985. 9261096 10.1074/jbc.272.34.20982

[8] Wang X, Collins HL, Ranalletta M, Fuki IV, Billheimer JT, et al (2007) Macrophage ABCA1 and ABCG1, but not SR-BI, promote macrophage reverse cholesterol transport in vivo. J Clin Invest 117: 2216–2224. 17657311 10.1172/JCI32057 PMC1924499

[9] Ji A, Meyer JM, Cai L, Akinmusire A, de Beer MC, et al (2011) Scavenger receptor SR-BI in macrophage lipid metabolism. Atherosclerosis 217: 106–112. 21481393 10.1016/j.atherosclerosis.2011.03.017 PMC3139003

[10] Liu T, Krieger M, Kan HY, Zannis VI (2002) The effects of mutations in helices 4 and 6 of ApoA-I on scavenger receptor class B type I (SR-BI)-mediated cholesterol efflux suggest that formation of a productive complex between reconstituted high density lipoprotein and SR-BI is required for efficient lipid transport. J Biol Chem 277: 21576–21584. 11882653 10.1074/jbc.M112103200

[11] Gu X, Trigatti B, Xu S, Acton S, Babitt J, et al (1998) The efficient cellular uptake of high density lipoprotein lipids via scavenger receptor class B type I requires not only receptor-mediated surface binding but also receptor-specific lipid transfer mediated by its extracellular domain. J Biol Chem 273: 26338–26348. 9756864 10.1074/jbc.273.41.26338

[12] Connelly MA, de la Llera-Moya M, Monzo P, Yancey PG, Drazul D, et al (2001) Analysis of chimeric receptors shows that multiple distinct functional activities of scavenger receptor, class B, type I (SR-BI), are localized to the extracellular receptor domain. Biochemistry 40: 5249–5259. 11318648 10.1021/bi002825r

[13] Connelly MA, De La Llera-Moya M, Peng Y, Drazul-Schrader D, Rothblat GH, et al (2003) Separation of lipid transport functions by mutations in the extracellular domain of scavenger receptor class B, type I. J Biol Chem 278: 25773–25782. 12730208 10.1074/jbc.M302820200

[14] Parathath S, Sahoo D, Darlington YF, Peng Y, Collins HL, et al (2004) Glycine 420 near the C-terminal transmembrane domain of SR-BI is critical for proper delivery and metabolism of high density lipoprotein cholesteryl ester. J Biol Chem 279: 24976–24985. 15060063 10.1074/jbc.M402435200

[15] Papale GA, Nicholson K, Hanson PJ, Pavlovic M, Drover VA, et al (2010) Extracellular hydrophobic regions in scavenger receptor BI play a key role in mediating HDL-cholesterol transport. Arch Biochem Biophys 496: 132–139. 20219439 10.1016/j.abb.2010.02.011 PMC2853188

[16] Guo L, Chen M, Song Z, Daugherty A, Li XA (2011) C323 of SR-BI is required for SR-BI-mediated HDL binding and cholesteryl ester uptake. J Lipid Res 52: 2272–2278. 21917726 10.1194/jlr.M019091 PMC3220294

[17] Papale GA, Hanson PJ, Sahoo D (2011) Extracellular disulfide bonds support scavenger receptor class B type I-mediated cholesterol transport. Biochemistry 50: 6245–6254. 21675794 10.1021/bi2005625 PMC3940413

[18] Ji Y, Wang N, Ramakrishnan R, Sehayek E, Huszar D, et al (1999) Hepatic scavenger receptor BI promotes rapid clearance of high density lipoprotein free cholesterol and its transport into bile. J Biol Chem 274: 33398–33402. 10559220 10.1074/jbc.274.47.33398

[19] Ueda Y, Royer L, Gong E, Zhang J, Cooper PN, et al (1999) Lower plasma levels and accelerated clearance of high density lipoprotein (HDL) and non-HDL cholesterol in scavenger receptor class B type I transgenic mice. J Biol Chem 274: 7165–7171. 10066776 10.1074/jbc.274.11.7165

[20] Trigatti BL, Rigotti A, Braun A (2000) Cellular and physiological roles of SR-BI, a lipoprotein receptor which mediates selective lipid uptake. Biochim Biophys Acta 1529: 276–286. 11111095 10.1016/s1388-1981(00)00154-2

[21] Kozarsky KF, Donahee MH, Rigotti A, Iqbal SN, Edelman ER, et al (1997) Overexpression of the HDL receptor SR-BI alters plasma HDL and bile cholesterol levels. Nature 387: 414–417. 9163428 10.1038/387414a0

[22] Webb NR, de Beer MC, Asztalos BF, Whitaker N, van der Westhuyzen DR, et al (2004) Remodeling of HDL remnants generated by scavenger receptor class B type I. J Lipid Res 45: 1666–1673. 15210842 10.1194/jlr.M400026-JLR200

[23] Rigotti A, Trigatti BL, Penman M, Rayburn H, Herz J, et al (1997) A targeted mutation in the murine gene encoding the high density lipoprotein (HDL) receptor scavenger receptor class B type I reveals its key role in HDL metabolism. Proc Natl Acad Sci U S A 94: 12610–12615. 9356497 10.1073/pnas.94.23.12610 PMC25055

[24] Murao K, Terpstra V, Green SR, Kondratenko N, Steinberg D, et al (1997) Characterization of CLA-1, a human homologue of rodent scavenger receptor BI, as a receptor for high density lipoprotein and apoptotic thymocytes. J Biol Chem 272: 17551–17557. 9211901 10.1074/jbc.272.28.17551

[25] Acton S, Osgood D, Donoghue M, Corella D, Pocovi M, et al (1999) Association of polymorphisms at the SR-BI gene locus with plasma lipid levels and body mass index in a white population. Arterioscler Thromb Vasc Biol 19: 1734–1743. 10397692 10.1161/01.atv.19.7.1734

[26] Hsu LA, Ko YL, Wu S, Teng MS, Peng TY, et al (2003) Association between a novel 11-base pair deletion mutation in the promoter region of the scavenger receptor class B type I gene and plasma HDL cholesterol levels in Taiwanese Chinese. Arterioscler Thromb Vasc Biol 23: 1869–1874. 12816880 10.1161/01.ATV.0000082525.84814.A9

[27] West M, Greason E, Kolmakova A, Jahangiri A, Asztalos B, et al (2009) Scavenger receptor class B type I protein as an independent predictor of high-density lipoprotein cholesterol levels in subjects with hyperalphalipoproteinemia. J Clin Endocrinol Metab 94: 1451–1457. 19158204 10.1210/jc.2008-1223 PMC2682469

[28] Brunham LR, Tietjen I, Bochem AE, Singaraja RR, Franchini PL, et al (2011) Novel mutations in scavenger receptor BI associated with high HDL cholesterol in humans. Clin Genet 79: 575–581. 21480869 10.1111/j.1399-0004.2011.01682.x

[29] Connelly MA, Klein SM, Azhar S, Abumrad NA, Williams DL (1999) Comparison of class B scavenger receptors, CD36 and scavenger receptor BI (SR-BI), shows that both receptors mediate high density lipoprotein-cholesteryl ester selective uptake but SR-BI exhibits a unique enhancement of cholesteryl ester uptake. J Biol Chem 274: 41–47. 9867808 10.1074/jbc.274.1.41

[30] Lowry OH, Rosebrough NJ, Farr AL, Randall RJ (1951) Protein measurement with the Folin phenol reagent. J Biol Chem 193: 265–275. 14907713

[31] Swarnakar S, Temel RE, Connelly MA, Azhar S, Williams DL (1999) Scavenger receptor class B, type I, mediates selective uptake of low density lipoprotein cholesteryl ester. J Biol Chem 274: 29733–29739. 10514447 10.1074/jbc.274.42.29733

[32] Oram JF, Yokoyama S (1996) Apolipoprotein-mediated removal of cellular cholesterol and phospholipids. J Lipid Res 37: 2473–2491. 9017501

[33] Duong M, Collins HL, Jin W, Zanotti I, Favari E, et al (2006) Relative contributions of ABCA1 and SR-BI to cholesterol efflux to serum from fibroblasts and macrophages. Arterioscler Thromb Vasc Biol 26: 541–547. 16410457 10.1161/01.ATV.0000203515.25574.19

[34] de la Llera-Moya M, Rothblat GH, Connelly MA, Kellner-Weibel G, Sakr SW, et al (1999) Scavenger receptor BI (SR-BI) mediates free cholesterol flux independently of HDL tethering to the cell surface. J Lipid Res 40: 575–580. 10064746

[35] Yancey PG, Bortnick AE, Kellner-Weibel G, de la Llera-Moya M, Phillips MC, et al (2003) Importance of different pathways of cellular cholesterol efflux. Arterioscler Thromb Vasc Biol 23: 712–719. 12615688 10.1161/01.ATV.0000057572.97137.DD

[36] Kellner-Weibel G, de La Llera-Moya M, Connelly MA, Stoudt G, Christian AE, et al (2000) Expression of scavenger receptor BI in COS-7 cells alters cholesterol content and distribution. Biochemistry 39: 221–229. 10625497 10.1021/bi991666c

[37] Sahoo D, Darlington YF, Pop D, Williams DL, Connelly MA (2007) Scavenger receptor class B Type I (SR-BI) assembles into detergent-sensitive dimers and tetramers. Biochim Biophys Acta 1771: 807–817. 16624615 10.1016/j.bbalip.2006.03.003

[38] Landschulz KT, Pathak RK, Rigotti A, Krieger M, Hobbs HH (1996) Regulation of scavenger receptor, class B, type I, a high density lipoprotein receptor, in liver and steroidogenic tissues of the rat. J Clin Invest 98: 984–995. 8770871 10.1172/JCI118883 PMC507514

[39] Reaven E, Cortez Y, Leers-Sucheta S, Nomoto A, Azhar S (2004) Dimerization of the scavenger receptor class B type I: formation, function, and localization in diverse cells and tissues. J Lipid Res 45: 513–528. 14657200 10.1194/jlr.M300370-JLR200

[40] Gaidukov L, Nager AR, Xu S, Penman M, Krieger M (2011) Glycine dimerization motif in the N-terminal transmembrane domain of the high density lipoprotein receptor SR-BI required for normal receptor oligomerization and lipid transport. J Biol Chem 286: 18452–18464. 21454587 10.1074/jbc.M111.229872 PMC3099662

[41] Rodrigueza WV, Thuahnai ST, Temel RE, Lund-Katz S, Phillips MC, et al (1999) Mechanism of scavenger receptor class B type I-mediated selective uptake of cholesteryl esters from high density lipoprotein to adrenal cells. J Biol Chem 274: 20344–20350. 10400657 10.1074/jbc.274.29.20344

[42] Ramjeesingh M, Huan LJ, Garami E, Bear CE (1999) Novel method for evaluation of the oligomeric structure of membrane proteins. Biochem J 342 Pt 1: 119–123. 10432308 PMC1220444

[43] Vinals M, Xu S, Vasile E, Krieger M (2003) Identification of the N-linked glycosylation sites on the high density lipoprotein (HDL) receptor SR-BI and assessment of their effects on HDL binding and selective lipid uptake. J Biol Chem 278: 5325–5332. 12429731 10.1074/jbc.M211073200

[44] Gavel Y, von Heijne G (1990) Sequence differences between glycosylated and non-glycosylated Asn-X-Thr/Ser acceptor sites: implications for protein engineering. Protein Eng 3: 433–442. 2349213 10.1093/protein/3.5.433 PMC7529082

[45] Braun A, Trigatti BL, Post MJ, Sato K, Simons M, et al (2002) Loss of SR-BI expression leads to the early onset of occlusive atherosclerotic coronary artery disease, spontaneous myocardial infarctions, severe cardiac dysfunction, and premature death in apolipoprotein E-deficient mice. Circ Res 90: 270–276. 11861414 10.1161/hh0302.104462

[46] Arai T, Wang N, Bezouevski M, Welch C, Tall AR (1999) Decreased atherosclerosis in heterozygous low density lipoprotein receptor-deficient mice expressing the scavenger receptor BI transgene. J Biol Chem 274: 2366–2371. 9891004 10.1074/jbc.274.4.2366

[47] Ueda Y, Gong E, Royer L, Cooper PN, Francone OL, et al (2000) Relationship between expression levels and atherogenesis in scavenger receptor class B, type I transgenics. J Biol Chem 275: 20368–20373. 10751392 10.1074/jbc.M000730200

[48] Zhang Y, Da Silva JR, Reilly M, Billheimer JT, Rothblat GH, et al (2005) Hepatic expression of scavenger receptor class B type I (SR-BI) is a positive regulator of macrophage reverse cholesterol transport in vivo. J Clin Invest 115: 2870–2874. 16200214 10.1172/JCI25327 PMC1236682

[49] Gu XJ, Kozarsky K, Krieger M (2000) Scavenger receptor class B, type I-mediated [H-3]cholesterol efflux to high and low density lipoproteins is dependent on lipoprotein binding to the receptor. Journal of Biological Chemistry 275: 29993–30001. 11001950 10.1074/jbc.275.39.29993

[50] Vergeer M, Korporaal SJ, Franssen R, Meurs I, Out R, et al (2011) Genetic variant of the scavenger receptor BI in humans. N Engl J Med 364: 136–145. 21226579 10.1056/NEJMoa0907687

[51] Adorni MP, Zimetti F, Billheimer JT, Wang N, Rader DJ, et al (2007) The roles of different pathways in the release of cholesterol from macrophages. J Lipid Res 48: 2453–2462. 17761631 10.1194/jlr.M700274-JLR200

[52] Van Eck M, Bos IS, Hildebrand RB, Van Rij BT, Van Berkel TJ (2004) Dual role for scavenger receptor class B, type I on bone marrow-derived cells in atherosclerotic lesion development. Am J Pathol 165: 785–794. 15331403 10.1016/S0002-9440(10)63341-X PMC1618614

[53] Yancey PG, Jerome WG, Yu H, Griffin EE, Cox BE, et al (2007) Severely altered cholesterol homeostasis in macrophages lacking apoE and SR-BI. J Lipid Res 48: 1140–1149. 17299204 10.1194/jlr.M600539-JLR200

[54] Yvan-Charvet L, Pagler TA, Wang N, Senokuchi T, Brundert M, et al (2008) SR-BI inhibits ABCG1-stimulated net cholesterol efflux from cells to plasma HDL. J Lipid Res 49: 107–114. 17960026 10.1194/jlr.M700200-JLR200

[55] Jian B, de la Llera-Moya M, Ji Y, Wang N, Phillips MC, et al (1998) Scavenger receptor class B type I as a mediator of cellular cholesterol efflux to lipoproteins and phospholipid acceptors. J Biol Chem 273: 5599–5606. 9488688 10.1074/jbc.273.10.5599

[56] Imjeti NS, Lebreton S, Paladino S, de la Fuente E, Gonzalez A, et al (2011) N-Glycosylation instead of cholesterol mediates oligomerization and apical sorting of GPI-APs in FRT cells. Mol Biol Cell 22: 4621–4634. 21998201 10.1091/mbc.E11-04-0320 PMC3226479

[57] Ishmael SS, Ishmael FT, Jones AD, Bond JS (2006) Protease domain glycans affect oligomerization, disulfide bond formation, and stability of the meprin A metalloprotease homo-oligomer. J Biol Chem 281: 37404–37415. 17040911 10.1074/jbc.M602769200

[58] Huseby ES, Crawford F, White J, Marrack P, Kappler JW (2006) Interface-disrupting amino acids establish specificity between T cell receptors and complexes of major histocompatibility complex and peptide. Nat Immunol 7: 1191–1199. 17041605 10.1038/ni1401

[59] Jang DS, Cha HJ, Cha SS, Hong BH, Ha NC, et al (2004) Structural double-mutant cycle analysis of a hydrogen bond network in ketosteroid isomerase from Pseudomonas putida biotype B. Biochem J 382: 967–973. 15228388 10.1042/BJ20031871 PMC1133972

[60] Ashida A, Yamamoto D, Nakakura H, Matsumura H, Uchida S, et al (2007) A case of nephrogenic diabetes insipidus with a novel missense mutation in the AVPR2 gene. Pediatr Nephrol 22: 670–673. 17216256 10.1007/s00467-006-0388-8

[61] Feys HB, Pareyn I, Vancraenenbroeck R, De Maeyer M, Deckmyn H, et al (2009) Mutation of the H-bond acceptor S119 in the ADAMTS13 metalloprotease domain reduces secretion and substrate turnover in a patient with congenital thrombotic thrombocytopenic purpura. Blood 114: 4749–4752. 19786614 10.1182/blood-2009-07-230615

[62] Wang Y, Maegawa S, Akiyama Y, Ha Y (2007) The role of L1 loop in the mechanism of rhomboid intramembrane protease GlpG. J Mol Biol 374: 1104–1113. 17976648 10.1016/j.jmb.2007.10.014 PMC2128867

[63] Hauser M, Kauffman S, Lee BK, Naider F, Becker JM (2007) The first extracellular loop of the Saccharomyces cerevisiae G protein-coupled receptor Ste2p undergoes a conformational change upon ligand binding. J Biol Chem 282: 10387–10397. 17293349 10.1074/jbc.M608903200

[64] Kyte J, Doolittle RF (1982) A simple method for displaying the hydropathic character of a protein. J Mol Biol 157: 105–132. 7108955 10.1016/0022-2836(82)90515-0

[65] Cao G, Garcia CK, Wyne KL, Schultz RA, Parker KL, et al (1997) Structure and localization of the human gene encoding SR-BI/CLA-1. Evidence for transcriptional control by steroidogenic factor 1. J Biol Chem 272: 33068–33076. 9407090 10.1074/jbc.272.52.33068

[66] Joy T, Hegele RA (2008) Is raising HDL a futile strategy for atheroprotection? Nat Rev Drug Discov 7: 143–155. 18239670 10.1038/nrd2489

[67] Wild S, Byrne CD (2008) Time to rethink high-density lipoprotein? Heart 94: 692–694. 18480346 10.1136/hrt.2007.126326

[68] Stylianou IM, Bauer RC, Reilly MP, Rader DJ (2012) Genetic basis of atherosclerosis: insights from mice and humans. Circ Res 110: 337–355. 22267839 10.1161/CIRCRESAHA.110.230854 PMC3357004

[69] Athyros VG, Katsiki N, Karagiannis A, Mikhailidis DP (2012) Should raising high-density lipoprotein cholesterol be a matter of debate? J Cardiovasc Med (Hagerstown) 13: 254–259. 22367577 10.2459/JCM.0b013e3283522422

[70] Hewing B, Fisher EA (2012) Rationale for cholesteryl ester transfer protein inhibition. Curr Opin Lipidol 10.1097/MOL.0b013e328353ef1d PMC3924318 22517614

[71] Degoma EM, Rader DJ (2011) Novel HDL-directed pharmacotherapeutic strategies. Nat Rev Cardiol 8: 266–277. 21243009 10.1038/nrcardio.2010.200 PMC3315102

